# Editorial: Human decision-making behaviors in engineering and management: A neuropsychological perspective

**DOI:** 10.3389/fnins.2022.1062171

**Published:** 2022-11-11

**Authors:** Hanliang Fu, Gui Ye, Jiayu Chen, Pin-Chao Liao

**Affiliations:** ^1^School of Management, Xi'an University of Architecture and Technology, Xi'an, China; ^2^Laboratory of Neuromanagement in Engineering, Xi'an University of Architecture and Technology, Xi'an, China; ^3^School of Management Science and Real Estate, Chongqing University, Chongqing, China; ^4^The International Research Center for Sustainable Built Environment, Chongqing University, Chongqing, China; ^5^Department of Construction Management, Tsinghua University, Beijing, China

**Keywords:** neuromanagement, human decision-making behavior, engineering management, psychological characteristics, neuropsychological perspective

Due to volatile, uncertain, complex, and ambiguous (VUCA) project context, human decision-making behaviors can affect the performance of engineering project. Therefore, understanding the process of perception, cognition, and decision caters to minimizing the risks that attribute to behaviors (Albert et al., 2020). Although traditional psychological/self-reporting approaches (e.g., survey, structured interview, etc.) are cost-effective, they have been criticized for the distortion caused by social desirability and subjectivity, which weakens the reproducibility and robustness of scientific findings. Recent development in physiological and neuropsychological techniques (e.g., EEG, fNIRS, eye-tracking, etc.), which deciphers the signals of the central nerve system, furnishing opportunities to untangle the mechanism of human decision-making behavior. These approaches can further promote the project performance in planning, organizing, executing, and ensure the success of engineering project. To acquire the focus of such research domain, this Research Topic focuses on human decision-making behaviors and management in engineering from a neuropsychological perspective. Seventeen of the twenty-two papers submitted to the journal were considered suitable for publication after a thorough double-blind peer-review process. The following is a summary of the key research findings of these research works.

Amongst the non-original research articles, Suomala and Kauttonen explained human behaviors and contextual decisions by applying intuitive models and Bayesian inference principles. Besides, they elaborate how behaviors arise in biological systems and how a better understanding of this biological system can lead to advances in the development of human-like AI. Three other review studies of neuroscience tools used in the areas of building construction and driving safety. Cheng et al. systematically reviewed the issues of eye-tracking research on construction hazard recognition, including participant selection, experiment design, device parameters, and analytical techniques (e.g., feature extraction, performance metrics, etc.). Peng et al. focused on safety behaviors in transportation systems. They introduced theories of electroencephalogram (EEG) and the utilization of EEG in scenarios of fatigued driving, distracted driving, and emotional driving. Lastly, Wang et al. utilized bibliometric methods to summarize the interdisciplinary applications of neuroscience tools in building construction. The results show that EEG and eye-tracking techniques are the predominant neuroscience tools in building construction studies, while functional near-infrared spectroscopy (fNIRS), functional magnetic resonance imaging (fMRI), and trigeminal nerve stimulation (TNS) are still at the adoption stage (Wang et al.).

Thirteen original research articles published within the Research Topic use various neuroscience tools [e.g., EEG, eye tracking, fNIRS, transcranial direct current stimulation (tDCS), etc.] to promote management efficiency in the engineering industry. EEG, for instance, has been used in five of all thirteen original research articles. Among them, Fan C. et al. regard EEG signals as the gold standard of cognitive status and proposed an end-to-end brain-computer interface framework named EEG-TNet for mental workload estimation of workers. The event-related potential (ERP) is a kind of real-time EEG signal related to the stimuli events (Chen et al., 2022). Therefore, ERP analysis techniques are widely used to explore the neural mechanisms of decision-making behaviors (Fu et al., 2022). Liu et al. from the Laboratory of Neuromanagement in Engineering, Xi'an University of Architecture and Technology, explored the public stereotypes of owning and renting a house by designing an ERP experiment. Zhang et al. conducted an ERP experiment to investigate the influence of the building narrative on individuals' approach-avoidance responses toward the stadium and the corresponding neural correlates. Hu et al. applied the ERP method to examine the effect of safety signs on cognitive control by taking the monitoring ability of conflicts and errors into account. Qian et al. proposed a lightweight multimodal cognition-aware computing framework integrating EEG, eye-tracking, heart rate, and video data under the VUCA environment. The multimodal cognition-aware computing framework provides specific technological solutions for the potential troubles of utilizing multimodal physiological and neuropsychological techniques in open VUCA environments.

Two studies using eye-movement parameters were presented in the Research Topic. Qu et al. proposed a method to identify human-computer interaction intention and cognitive state based on eye movement and EEG parameters to solve the unsmooth and inefficient problems in human-computer interaction. Equipment teleoperation is a powerful solution for hazardous construction environments and attracted researchers' attention. Fan J. et al. investigated the effect of eye movement on teleoperation performance in excavating tasks. They built eye-tracking heatmaps with virtual annotations, representing operators' visual attention allocation. Based on these heatmaps, they concluded that visual attention is influenced by the excavating tasks and the shape of virtual annotations plays a critical role in visual attention allocation.

The association between engineering decision-making and neuro-cognition was examined in two studies using fNIRS. Hu and Shealy  used fNIRS to measure the neuro-cognition changes of the engineers when they were asked to evaluate the implementation of conventional stormwater and green stormwater design options. So as to test whether design engineers think about the environmental and social sustainability benefits of green infrastructure can influence what attributes engineers consider and how they weigh these attributes during the design decision-making process. Ding et al. focused on the problem of the increasing hazards caused by construction and demolition (C&D) waste in the construction industry. In order to explore measures that could mitigate the problem, fNIRS has been used to investigate whether the media can influence consumers' willingness to pay for C&D waste recycling products and thus increase consumers' choices.

Yang et al. attempted to explore the role of the right temporoparietal junction in the effects of the CEO-to-employee pay ratio on potential investors' perceived investment potential in the construction industry. The mechanisms underlying these effects of tDCS in the right temporoparietal junction on the perceived investment potential are also explored in the study.

Lastly, three papers in this Research Topic use different tools to explore the measures to increase the efficiency of engineering education and the construction industry. Zhao, Ao, et al. found that the experience of home-based learning significantly influenced the attitudes of university students of civil engineering, which in turn had a positive influence on their intention to continue online education. Chen et al. developed a whole-process digital management system platform and used a variety of advanced digital construction hardware and three-dimensional model software to coordinate the measurement, design, construction, and inspection work in the process of highway engineering construction. Zhao, Wang, et al. explored the key influencing factors of urban intelligent transportation construction and proposed a root cause analysis method based on a fuzzy cognitive map to model the construction process.

We appreciate the contributions of all the authors, the reviewers, and the editorial board members for contributing to this Research Topic. We hope that this Frontiers Research Topic can enrich the body of knowledge and encourage the utilization of cognitive neuroscience theories, methods, and tools in the Architecture, Engineering, and Construction Industries.

## Author contributions

All authors listed have made a substantial, direct, and intellectual contribution to the work and approved it for publication.

## Conflict of interest

The authors declare that the research was conducted in the absence of any commercial or financial relationships that could be construed as a potential conflict of interest.

## Publisher's note

All claims expressed in this article are solely those of the authors and do not necessarily represent those of their affiliated organizations, or those of the publisher, the editors and the reviewers. Any product that may be evaluated in this article, or claim that may be made by its manufacturer, is not guaranteed or endorsed by the publisher.
